# The Mental State of Hystericals

**Published:** 1902-08

**Authors:** 


The Mental State of Hystericals. A Study of Mental Stigmata and Mental Accidents. By Pierre Janet, Litt. D., M. D., Professor of Philosophy at the College Rollin. With a preface by Professor J. M. Charcot. Translated by Caroline Rollin Corson. Octavo, pp. 553. New York and London: G. P. Putnam's Sons. 1902.

Charcot had the happy faculty of surrounding himself with young men who like himself were strongly imbued with medical science and medical philosophy. Of the former such names as Marie, Raymond, Féré, Brissau and Joffroy, and of the latter, Pierre Janet and Tourette, are the best known and their writings bear the stamp of the master's mind behind them all.

It remained for Janet to produce a work after Charcot's own heart, a subject which both gave unusual attention and treated with unusual skill. Up to Charcot's time hysteria was an unknown field full of superstition and ignorance; gradually he converted it into a field full of the highest type of research and thought and Janet's work of today is only a sample of what that field has brought forth.

The author has divided the work into two parts, part I. dealing with mental stigmata; part II. with mental accidents. Hysteria, according to Briquet, is a general disease which modifies the whole organism. If it disturbs nutrition and all the physiological functions, it disturbs also the psychological phenomena, which are among the functions of the brain. It is these psychological perturbations which the author proposes to examine in this work.

The anesthesias or insensibilities, so common among hystericals, are dealt with very thoroughly by the author and their explanation is left a blank. The author then considers the amnesias, the abulias, motor disturbances and modifications of the character in hystericals. These chapters bear evidence of close study and sound reasoning. The mental accidents comprise the grosser hysterical manifestations as somnambulism attacks, delirium, suggestion and subconscious acts and fixed ideas.

The concluding chapter deals with hysteria from a psychological point of view and the author shows it to be a malady by representation. "No definition of hysteria has ever been given and never will be given" says Laseque, and the author wisely refrains from attempting one—although he gathers up his conclusions as follows: Hysteria is a form of mental disintegration characterised by a tendency toward the permanent and complete undoubling of the personality.

The author found that heredity plays a very prominent part in hysteria; and a critical age—not exactly the physical age of puberty so much as the moral puberty, which comes somewhat later and plays such a preponderant rôle.

To the neurologist, alienist, psychologist and medical philosopher, the book is invaluable, while to the progressive physician it will reveal a great light on this important subject.

The translation has been excellently rendered by Mrs. Corson of Cornell University and its English is as finished as is the subject matter in its original. W. C. K.

